# Chemiluminescent microparticle immunoassay based detection and prevalence of HCV infection in district Peshawar Pakistan

**DOI:** 10.1186/1743-422X-11-127

**Published:** 2014-07-12

**Authors:** Muhammad Ilyas, Irshad Ahmad

**Affiliations:** 1 Department of Microbiology and Biotechnology, Sarhad University of Science and Information Technology (SUIT), 25120 Peshawar, Pakistan; 2 Biology Department, King Fahd University of Petroleum and Minerals (KFUPM), 34464 Dhahran, Saudi Arabia

**Keywords:** HCV, CMIA, Prevalence

## Abstract

**Background:**

Due to the high rate of asymptomatic infections an advanced screening assay is of prompt importance to be used for the clinical diagnosis of HCV. Early detection of anti HCV is the first step in the management of chronic hepatitis and in the selection of patients needing treatments. In the current study we have first time used the advanced serological diagnostic technique i.e. Chemiluminescent Microparticle Immuno Assay (CMIA) for the detection of HCV infection in Peshawar Pakistan.

**Methods:**

A total number of 982 samples were collected among the general public belongs to the different areas of district Peshawar. The samples were centrifuged at high speed to obtain a clear supernatant serum. All the samples were run on Architect system a fully automated immuno analyzer CMIA base technology.

**Results:**

Out of 982 blood samples analyzed in this study, 160 (15.9%) were confirmed to be positive for active HCV infection. The overall prevalence was found to be 13.4%. Gender wise prevalence was recorded to be higher in male (19.1%) than female (12.7%). The age group 21-30 years was identified as the highest risk group among the studied population.

**Conclusion:**

Among the tested samples, overall prevalence of active HCV infection was found to be 13.4% in the general population of Peshawar Pakistan. The young middle aged population of this region was at higher risk of HCV ailments compared to the other age groups.

## Background

Hepatitis C is a viral infectious disease of liver. At early stages the infection is asymptomatic but once established, it can progress to advanced liver diseases such as liver fibrosis and ultimately cirrhosis. These liver diseases can further lead to other complications such as liver failure and liver cancer [1]. HCV is a plus-stranded RNA virus and is a distinct member of the family*Flaviviridae*. HCV has infected about 200 million people worldwide which are about 3.3% of the world population [2]. Approximately 20-30% of patients naturally clear the virus. About 70-80% acute HCV infections become chronic that leads to the development of cirrhosis in 20% of cases while the same percentage of patients becomes victim of hepatocellular carcinoma. Acute hepatitis C occurs during the first six months of HCV infection [3].

Prevalence of HCV varies throughout the world. Liver cirrhosis is a major cause of mortality and HCV related liver cancer is the 8^th^ common cancer worldwide [4,5]. In Pakistan, more than 10 million people are suffering from HCV that comprise 6% of total population, with high morbidity and mortality [6]. In previous studies high HCV prevalence was reported in other cities of Pakistan. There were 16% in Lahore, 20.6% in Faisalabad and 23.8% in Gujranwala [7] but no such report is there as far as Peshawar region is concerned.

Due to high rate of asymptomatic infections, an advanced screening assay is of prompt importance to be used for the clinical diagnosis of HCV. Early anti HCV detection is the first step in the management of chronic hepatitis in order to select the patients for timely treatment [8]. Various methods are implemented for the diagnosis of hepatitis infection i.e. Immune Chromatographic Technique (ICT), Enzyme Linked Immuno Sorrbant Assay (ELISA) and HCV-RNA by PCR, but due to the false positivity rate of HCV with ICT based methods, ELISA is considered to be more consistent than ICT based HCV diagnosis [9]. The detection of HCV RNA by PCR is more reliable; however, PCR assay is costly, has technical hitches and it needs skilled personnel for the operation and interpretation of the output. Patients in this region with low socioeconomic status will not be able to afford it. The current study we have focused on, the Chemiluminescent Microparticle Immuno Assay (CMIA), an advanced serological diagnostic technique, provides a rapid, cost-effective and reliable way to detect HCV, and therefore, is the method that was implemented for the diagnosis of hepatitis infection.

## Results and discussion

**Figure 1:**
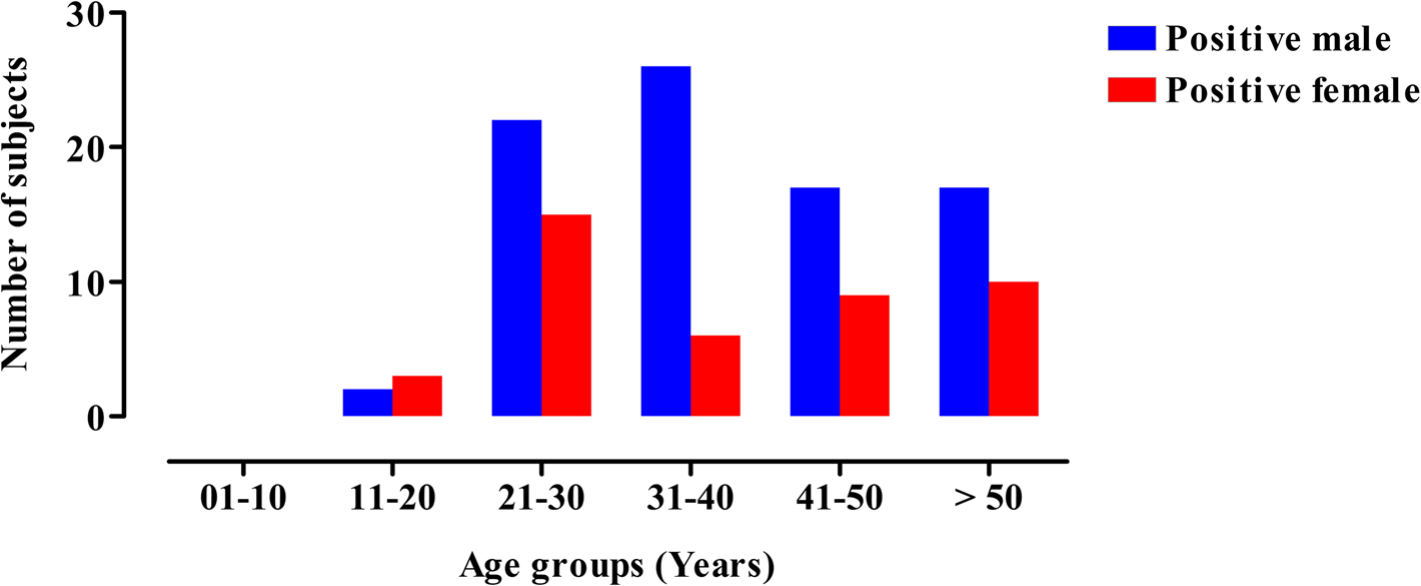
Prevalence of HCV positive samples in different age groups with respect to male/female population.

A total of 982 blood samples were collected from the district Peshawar Pakistan and were screened for anti HCV. The current study was carried out during February, 2013-14. Among 982 samples 127 were detected as HCV positive. Figure 1 shows a prevalence of HCV positive samples in different age groups with respect to male/female population. As shown in Table 1, the gender-wise prevalence of active HCV infection was estimated to be 15.4% in male (84 positive out of 543 samples) and 9.7% in female subjects (43 positive out of 439 samples) respectively. Considering the age group criterion, significant differences were observed in the prevalence of HCV in both genders. The highest prevalence of 18.5% was observed in age group 21-30 (*p* = 0.9407, OR = 0.972, 95% CI = 0.470-2.014), however statistically significant active HCV infection was noted in age group 31-40 with a prevalence of 17.2% (*p* = 0.0007, OR = 4.626, 95% CI = 1.802-11.88). Both of these values are higher compared to the age group 11–20 years having a prevalence of 3.7% (*p* = 0.5889, OR = 0.607, 95% CI = 0.098-3.761). The age groups of 41-50 and > 50 was associated with a decrease trend of active HCV prevalence of 13.1% (p = 0.1757, OR = 1.803, 95% CI = 0.761-4.268) and 14.6% (*p* = 0.2740, OR = 1.595, 95% CI = 0.687-3.701) respectively.

**Table 1:** Frequency of HCV positive samples among different age groups and overall prevalence

**Age groups (years)**	**Sample size**	**Male +/-**	**Female +/-**	***p *** **value**	**Odds ratio**	**Confidence interval**	**Overall prevalence (%)**
01-10	80	00/50	00/30	-	-	-	-
11-20	135	02/68	03/62	0.5889	0.607	0.098-3.761	3.7
21-30	200	22/98	15/65	0.9407	0.972	0.470-2.014	18.5
31-40	185	26/74	06/79	0.0007***	4.626	1.802-11.88	17.2
41-50	198	17/88	09/84	0.1757	1.803	0.761-4.268	13.1
> 50	184	17/81	10/76	0.2740	1.595	0.687-3.701	14.6
**Total**	**982**	**84/459**	**43/396**	**-**	**-**	**-**	**13.4**

Chi-square test was applied and *p* < 0.05 was considered as significant at 95% confidence interval.

***p = value is significant.

Previous reports on HCV prevalence in other regions of Khyber Pakhtunkhwa, Pakistan were shown in Table 2. A high prevalence of HCV (57% and 36%) was observed among the thalassaemic patients [10,11]. Others regions like Mardan, Risalpur, Peshawar, Abbottabad and Bunner showed HCV prevalence as 3.69%, 3.40%, 2.20%, 8% and 4.57% respectively.

**Table 2:** HCV prevalence in other regions of Khyber Pakhtunkhwa Pakistan

**Region**	**Method**	**Population size**	**HCV prevalence**	**Reference**
Mardan	ELISA	15550	3.69%	[12]
Risalpur	ELISA	2558	3.40%	[13]
Peshawar	MEIA	3430	2.20%	[14]
Abbotabad	ELISA	102	8%	[15]
Bunner	ELISA	16400	4.57%	[16]
Peshawar	ELISA	80	36%	[11]
NWFP(KPK)	ELISA	250	57%	[10]

Hepatitis C virus (HCV) is a major cause of liver disease and has a high potential to cause significant morbidity and mortality worldwide [17]. Prevalence of HCV in Pakistan is the highest in the world and estimated to be 4.8% [18]. Currently, about 10 million people are infected with HCV in Pakistan [19]. The prevalence of hepatitis C varies in different provinces of Pakistan. It was found to be high in Sind and Punjab that is 5-6% [20]. The present study was conducted during February, 2013-14 in order to find out the prevalence of HCV among the general public of district Peshawar, Pakistan.

We have found the overall prevalence of HCV as 13.4% which is higher than 4.9% previously reported among the general public of Lahore, Pakistan [1]. This major difference among the overall prevalence may be due to using of different diagnostic techniques in the laboratories, immunological status of the persons and lack of awareness regarding hepatitis C virus particularly in district Peshawar Pakistan. Another study conducted among the healthy donors in Bolichistan Pakistan reported 20.8% prevalence which was slightly higher than our present study [21]. This is due to the lack of education, poverty and unhygienic health status of the people in the region.

In the current study the incidence of HCV was higher in the age group 21-30 years i.e. 18.5% which is in accordance with the previously reported results [21]. High prevalence of HCV was reported to be 49.01% among the age group 21-25 years pregnant women in Sindh [22], which were higher than our present study, but the overall prevalence of hepatitis C virus was found to be as higher in the middle age group of the population in both studies.

The most terrible condition which we have observed in this study is the high prevalence of HCV in the young and middle aged people i.e. 21-30 and 31-40 years old respectively. A number of studies conducted on the prevalence of Hepatitis C virus in different areas of Pakistan [1,21] showed high incidence of Hepatitis C virus prevailed in these age groups. The occurrence of hepatitis among these age groups are contributed by lack of awareness, used of unsterile syringes, repeated use of razors and contaminated scissors for different customers without prior sterilization. It was observed that about 90% of barbers did not washed hands, 80% did not changed aprons and 66% did not changed towels after each customer [23].

Another bad practice which made the environments worse is the recycling of used syringes. A very young scavenger of waste products around 18 to 20 years of age sells 20-25 syringes per day to the health care waste dealers against money and the same child gets needle stick injury around none to three times per week [24].

The occurrence of hepatitis C virus found in the age group >50 years was 14.6% in the current study which is in accordance with the previously reported data [25]. The overall prevalence of active HCV among different aged groups reported in KPK, Pakistan was 7% by using ICT techniques [26] was much lower than our present study (13.4%). This disagreement is due to small sample size in the former study and unreliable serological diagnostic techniques (ICT) used having low specificity and sensitivity.

The frequent methods used for the diagnosis of HCV infection based on the detection of anti HCV antibodies in the serum or plasma, but nevertheless the concentration of these antibodies reaches to detectable level after a long window period of HCV infection. Additionally viruses eliminating from the blood but still their presence in the serum or plasma for a long period of time could not differentiate between current and past HCV infection. At the same time methods used like ELISA and CMIA for detection of anti HCV antibodies gives false positive results. These false positive results for anti HCV are likely in population having low HCV prevalence due to some cross reactivity of others viral antigen and antibodies in individual have immune disorder [27-29]. According to the CDC all the screening methods used for the detection of anti HCV need supplementary methods like HCV RNA or nucleic acid testing (NAT) for their further confirmation. In the current study the detection of anti HCV antibodies by CMIA techniques have some disadvantages of giving false positive results, therefore further tests like NAT to detect HCV RNA is needed. However in spite of these recommendations supplementary tests are not performed in many laboratories due to some reasons which include technical complexity of the methods, high cost, long procedures. In such condition the guidelines set issued by CDC recommendations including option to use a signal to cutoff ratios to limit the number of the samples needing supplemental testing. According to CDC guidelines [30] the FDA approved anti HCV screening kits (ELISA, CMIA and RIBA) establishes specific signals to cutoff ratio which is S/CO ≥5.0 for chemiluminescent immunoassay by architect system from Abbott diagnostic services which predicted a true antibodies positive results >95% of the time. In the present study positive results for the tested samples were obtained where serum index (S/CO ≥5.0).

In the present study anti HCV detection was done by using a very sensitive and more advance diagnostic technique CMIA on the stat of the art Architect system which is a fully automated immuno analyzer of Abbott diagnostic services.

## Conclusions

In this study we have used the most reliable, sensitive and advanced serological diagnostic technique for the detection of HCV antibodies in serum of infected persons. Among the tested samples, overall prevalence of active HCV infection was found to be 13.4% in the general population of Peshawar Pakistan. The HCV infection prevailed among all age groups but young middle age population of this region was at higher risk of HCV ailments compared to the other age groups. This study will be helpful to the health care policy makers to design strategies for controlling and eradication of Hepatitis C infection in Peshawar, Pakistan.

## Methods

### Sample collection

A total number of 982 sample were collected among the general public belongs to different areas of district Peshawar Pakistan. About 3 cc blood is collected from each individual in gel vaccutainer tube. The relevant demography of the persons was recorded in separate forms. The age groups included in this study was 01-80 years.

### Sample processing

The samples were centrifuged at high speed about 8000 rpm for 15 minutes in order to obtain a clear supernatant serum. All the samples were run on Architect system which is fully automated immuno analyzer of the Abbott diagnostic services.

### Chemiluminescent Microparticle Immuno Assay (CMIA)

Chemiluminescent Microparticle Immuno Assay is the modified and advanced form of the Enzyme Linked Immuno Sorrbant Assay (ELISA) technique. Architect system is designed to detect antibodies to putative structural and non structural protein (HCr-43, c-100, NS3, NS4) of HCV genome [31]. In the final reaction of Anti HCV detection, bound achridinylated conjugates were used to generate chemiluminescent signals. Results were obtained automatically by the software by comparing the chemiluminescent signals obtained from the reaction product of the sample with the signal of the cutoff value previously obtained by Anti HCV calibration. The overall specificity and sensitivity of this method is 99.6% and 99.7% respectively.

### Statistical analysis

Data was analyzed statistically by using GraphPad Prism 5 (GraphPad Software Inc. San Diego CA, USA). The Chi-square test was used to analyze the qualitative data. A *p* value < 0.05 was considered as significant. Odds ratio with 95% confidence interval was used to evaluate the association of various age groups with respect to HCV status of male/female population.

## Competing interests

The authors declare that they have no competing interests.

## Authors’ contributions

MI carried out samples collection and has participated in the experimental work. IA designed the overall study and prepared the manuscript. Both authors edited and approved the final manuscript.
